# Are Primary Health Care Visits Associated With Reduced Risk of Hospital Readmissions After Discharge From Geriatric Inpatient Departments? Evidence From Stockholm County

**DOI:** 10.1177/21501319241277413

**Published:** 2024-09-08

**Authors:** Mahwish Naseer, Carl Willers, Anne-Marie Boström, Amelie Lindh Mazya, Gunnar H Nilsson, Stefan Fors, Elisabeth Rydwik

**Affiliations:** 1 Karolinska Institutet, Stockholm, Sweden; 2 FOU nu, Research and Development Center for the Elderly, Region Stockholm, Järfälla, Sweden; 3 Karolinska University Hospital, Stockholm, Sweden; 4 Stockholms Sjukhem. Stockholm, Sweden; 5 Danderyd Hospital, Danderyd, Sweden; 6 Karolinska Institutet & Stockholm University, Stockholm, Sweden; 7 Centre for Epidemiology and Community Medicine, Region Stockholm, Stockholm, Sweden; 8 Stockholm University, Stockholm, Sweden

**Keywords:** older adults, readmissions, post-discharge care, primary care

## Abstract

**Introduction/objectives:**

Primary health care visits post-discharge could potentially play an important role in efforts of reducing hospital readmission. Focusing on a single or a particular type of visit obscures nuances in types of primary care contacts over time and fails to quantify the intensity of primary health care visits during the follow-up period. The aim of this study was to explore associations between the number and type of primary health care visits post-discharge and the risk of hospital readmission within 30 days.

**Methods:**

A register-based closed cohort study. The study population of 6135 individuals were residents of Stockholm who were discharged home from any of the 3 geriatric inpatient departments, excluding those who were readmitted within the next 24 h. The dependent variable was hospital readmission within 30 days of discharge. The key independent variable was the number and type of primary health care visits in 30 days post-discharge. Cox-regression with time-varying covariates was employed for data analyses.

**Results:**

Approximately, 12% of the participants were readmitted to hospital within 30 days. There was no statistically significant association between number of primary care visits post-discharge and readmission (HR 1.00; 95% CI 1.00-1.01). Compared to no primary health care visit, no statistically significant association were found for administrative care related visits (HR 0.33, 95%CI 0.08-1.33), clinic visits (HR 0.93, 95%CI 0.71-1.21), home visits (HR 1.03, 95%CI 0.84-1.27), or team visits (HR 0.76, 95%CI 0.54-1.07).

**Conclusions:**

There were no associations between primary health care visits post-discharge and hospital readmission after geriatric inpatient care. Further studies using survey or qualitative approaches can provide insights into the factors that are relevant to post-discharge care but are unavailable in this type of register data studies.

## Introduction

Hospital readmissions are commonly related to the quality of care, insufficient discharge planning, disease exacerbations or lack of self-management of illness at home.^1-3^ Although readmissions are generally costly for the healthcare systems, readmissions are also a burden for the patients and their families.^4,5^ Therefore, reducing avoidable readmissions has the potential to optimize quality of care and improve patient health outcomes.^3,5^ In research and practice, readmissions are commonly discussed as a potential trade-off between care utilization in the primary health care (PHC) and hospital.^1^ In this study, we explored whether PHC visits post-discharge is associated with lower risk of readmission among geriatric patients.

A post-discharge PHC visit provides an opportunity to discuss problems arising after hospital discharge and medicine reconciliation, potentially reducing the risk of readmissions.^6,7^ Therefore, PHC can lead efforts to reduce hospital readmission; however, there are few studies focusing on interventions arising from PHC.^8^ There is also a policy shift toward reinforcing PHC in many countries, for example, in Sweden the inquiry coordinated development for good quality local health care (SOU 2019: 29) was enacted in 2019 which proposed that PHC must be strengthened and access to care should be improved.^9,10^

Previous studies on health care visits post-discharge and readmission have led to mixed results. Post-discharge single home visits had no statistically significant association with hospital readmissions among individuals aged >65 years in Denmark.^11^ Yet, a Danish trial found protective effects of 3 visits (a mix of home and clinic visits) post-discharge by a team of GP and the district nurse and a reduced risk of readmissions among individuals aged ≥78 years discharged from geriatric or medical wards.^12^ Similarly, another intervention study including patients discharged from a Danish geriatric department found that the follow-up visit by a multidisciplinary geriatric team was associated with lower risk of hospital readmission only in patients discharged to the skilled nursing facility while no statistically significant association was seen among those discharged to their homes.^13^

Inconsistent methodologies concerning intensity of post-discharge care, type of contact (eg, home visit, team visit), patient characteristics and length of follow up period likely explain these diverging results. Patients in the geriatric departments are often afflicted with multiple medical conditions and are dependent in activities of daily living, hence have complex care needs. Accordingly, geriatric patients with severe health problems will likely need combined help from social and health care providers, post-discharge. Thus, it is expected that patients have several different types of PHC visits which may influence the risk of hospital readmissions. Focusing on a first or single PHC visit does not account for the change of type of PHC contact over time and fails to quantify the intensity of PHC visits during the follow-up period. To our knowledge, no study to date have considered the change in the type of PHC visits over time and readmissions among individuals in need of geriatric care.

This study aims to explore associations between the number and type of PHC care visits post-discharge and hospital readmission within 30 days.

## Material and Methods

### Study Design, Population, and Setting

This is a closed cohort study. The study population includes all the individuals who lived in the Stockholm County and were admitted to any of the 3 geriatric inpatient departments operated by the Stockholm Region during 2016 (N = 8082). Individuals discharged to institutions or ordinary homes may be eligible for different social and health care routines. Therefore, the analytical sample was restricted to the individuals discharged to their ordinary homes (N = 6148). Individuals readmitted within 24 h of discharge (N = 13) were excluded providing analytical sample n = 6135. If an individual had more than one admission to the geriatric inpatient departments during 2016, the last admission was used as the index admission in this study.

Stockholm county has approximately 2 440 000 inhabitants, and adults aged ≥65 years constitute 16.0% of the population. The provision of medical care including hospital care, outpatient care, and home health care is the responsibility of the Stockholm Region while the 26 municipalities within Stockholm Region are responsible for social care. Sweden has a universal and mainly tax financed healthcare system where users pay a small fraction of the total cost. All residents of Sweden are assigned to a PHC clinic, and have the right to choose their preferred PHC provider. In this study, 3 of 13 geriatric inpatient departments were included.^14^ In 2016 (ie, at the time of the data collection for this study) these geriatric inpatient departments were located at 3 different hospital facilities and the resources of these 3 geriatric departments were supposed to have been equivalent.

### Data Sources

Electronic health care records at the geriatric inpatient wards were used to obtain relevant information from the index admission. The data on healthcare utilization within 30 days of discharge from the index admission was retrieved from Stockholm Region’s administrative data warehouse (VAL). All health care providers, including hospital and outpatient care, are obliged to report data to the VAL. The patient level data was linked to the 2 national registers: the Longitudinal Integration Database for Health Insurance and Labour Market (LISA) for sociodemographic variables and the Social Services Register for home help services. These registers were linked via personal numbers (assigned to all individuals living in Sweden) that were encrypted. The Swedish Ethical Review Authority has granted ethical approval for this study (reg. no. 2018/247-32) and patient consent was not required.

### Variables

#### Dependent variable

The dependent variable was hospital readmission within 30 days (Yes/No)—the most studied time frame in the literature for measuring hospital readmission.^6,15^ A 30 days’ time frame reduces the risk of bias introduced by factors unrelated to index admission.^16^

#### Exposure variables

Post-discharge PHC utilization was measured as number of visits and type of visits in 30 days. In this study, all visits were included irrespective of care providers’ profession and type of contact. However, assistant nurse, nurse, physician, and physiotherapist are the professions commonly provide PHC to geriatric patients in next 6 months post-discharge.^14^ Information on “type of visit” was obtained from VAL database describing how the care was delivered. The variable “type of visit” was categorized into “no PHC visit,” “clinic visits (direct contact at PHC clinic),” “home visits (visit performed at patients’ home),” “team visits (visit by a team of different healthcare professionals),” and “administrative care related contacts (contacts of administrative character not providing medical advice).”

#### Covariates

Potential covariates were selected based on previous research on hospital readmission, the Andersen’s model of health care use and availability of data.^1,2,17,18^ Briefly, Andersen’s model proposes that explanatory factors of care use could be grouped into predisposing factors (eg, age, sex), enabling resources (eg, social support), and need factors (eg, health status).

*Sociodemographic factors* were age, sex, education, and living arrangements. Levels of education were based on number of schooling years and were categorized as primary (<9 years), lower secondary (9-10 years), upper secondary (2-3 years), post-secondary (1-3 years), and higher post-secondary (master degree or higher). Living arrangements were defined as cohabiting or living alone.

*Health related factors*: The information on diagnosis, medication, activities of daily living (ADL) and risk screening measures was obtained from electronic health care records at the index admission. The number of diagnosis (based on ICD 10 classification) at discharge was modeled as continuous variable. Polypharmacy was defined as ≥ 5 different medications prescribed at discharge.^19^ Barthel index was used to measure ADL such as walking, dressing, bathing. The score ranges from 0 to 100; high score indicates higher independence.^20^ Barthel index was modeled as continuous variable. Three risk screening measures (Mini Nutritional Assessment-short form (MNA-SF), the Downton Fall Risk Index, and the Norton pressure ulcer risk screening) were included and modeled as binary variables. The cut-off levels are based on previous research; risk of malnutrition (MNA ≤11^21^), high risk of fall (Downton ≥3,^22^ and risk of developing pressure ulcer (Norton ≤20^23^).

*Care related factors* included length of stay of index admission, number of specialist care visits (including emergency department visits) and hours of home help services granted in the 30 days post-discharge. All care related factors were modeled as continuous variables.

### Data Analyses

Observational studies exploring associations between PHC visits and hospital readmission face several challenges. Health status at the index admission can affect the intensity and type of PHC visits post-discharge, the probability of PHC visits can change over time, and readmission early after discharge can impact the probability of receiving PHC.^16^ In our study, 13% of the patients received their first PHC visit on the same day and 57% of the patients within 3 days of their discharge. To avoid biases related to exposure time,^24^ individuals readmitted within 24 h of discharge were excluded (n = 13), time to mortality was censored, and the exposure was modeled as a time-varying covariate.

Descriptive analyses were performed for all the independent variables and presented separately for readmitted and not readmitted respectively. Continuous variables were described by mean and standard deviation, and categorical variables were summarized by absolute and relative frequencies. For categorical data, difference between groups was assessed using Chi test, whereas for continuous data, difference between groups was evaluated using *T*-test.

Cox regression with time varying covariate was employed to estimate the associations between PHC visits post-discharge and hospital readmission.^25^ Results are presented as hazard ratios (HRs) with 95% confidence intervals (CIs). For all the analyses, Statistical software R version 4.3.1 was used.

## Results

### Characteristics of the Study Sample

The mean age of the participants was 82.9 years and 63.7% were women (Table 1). The study sample consisted of 6135 patients of whom 12.8% got readmitted within 30 days of discharge. No statistically significant differences were observed for sociodemographic factors between those who were readmitted and not readmitted apart from sex (*P* < .001).

**Table 1.** The Baseline Characteristics of All the Study Participants, Stratified by Hospital Readmission in 30 Days.

	All	No readmission	Readmission	*P*-value
Variables	(N = 6135)	(N = 5352)	(N = 783)	
*Sociodemographic factors*				
Age Mean (SD)	82.9 (8.13)	82.9 (8.11)	82.9 (8.32)	.973
Women %	63.7	64.8	55.7	<.001
Education %				.296
Primary	21.2	21.3	20.6	
Lower secondary	13.1	13.3	11.6	
Upper secondary	36.3	36.0	37.9	
Post secondary	19.9	20.0	18.9	
Higher post-secondary	9.5	9.3	11.1	
Living alone %	59.9	59.9	59.8	.925
*Health related factors*				
Number of diagnoses Mean (SD)	4.52 (1.76)	4.46 (1.75)	4.94 (1.79)	<.001
Polypharmacy (≥5 drugs) %	83.1	82.5	87.7	<.001
Physical function				
Barthel index, Mean (SD)	57.3 (25.8)	57.4 (25.7)	56.8 (26.3)	.538
Risk of malnutrition (MNA-SF ≤ 11) %	79.8	79.4	82.9	.044
Risk of fall, Downtown ≥ 3%	83.8	83.4	86.6	.025
Risk of pressure ulcer, Norton ≤ 20%	20.5	19.8	25.3	<.001
*Care related factors*				
Length of stay at index admission, Mean (SD)	8.44 (5.00)	8.36 (4.92)	9.02 (5.47)	.001
Number of specialist care visits, Mean (SD)	2.16 (7.18)	2.04 (7.26)	3.01 (6.51)	<.001
Home help (hours), Mean (SD)	56.7 (129)	57.4 (131.5)	51.9 (111.4)	.215
*Primary health care visits*				
Number of primary care visits per day				
Mean (SD)	0.33 (0.49)	0.32 (0.49)	0.39 (0.53)	<.001

Living alone and risk screening measures have <1.7% missing observations. Level of education has 3% and Barthel index has 9% missing observations.

The mean number of diagnoses was 4.52 and 83.1% of the sample had polypharmacy. The mean score of Barthel index was 57.3. In risk screening measures, 79.8% of the sample was at risk of malnutrition, 83.8% had risk of fall, and 20.5% had risk of pressure ulcer. Individuals who were readmitted within 30 days had statistically significantly higher mean of number of diagnoses (*P* < .001), polypharmacy (*P* < .001), risk of malnutrition (*P* = .044), risk of fall (*P* = .025), and risk of pressure ulcer (*P* < .001) at the index admission than those who were not readmitted.

Regarding care related factors, the mean length of stay of index admission and mean number of specialist care visits were statistically significantly higher in the readmitted group (*P* = .001). No statistically signification difference was observed in the mean number of home help hours.

**Figure 1.**
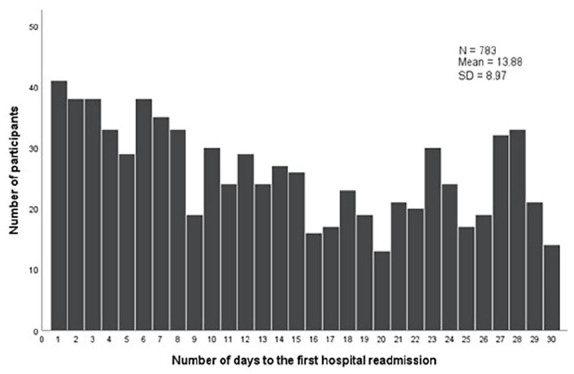
Number of days to the first hospital admission in 30 days (N = 783).

Figure 1 displays number of days to the readmission and the mean number of days to the readmission was 13.9.

### Descriptive Analyses of PHC Visits Post-Discharge

The mean number of PHC visits for the study sample was 0.33. The mean number of visits per day were significantly higher in the readmitted group (0.39) than those who were not readmitted (0.32, *P* < .001; Table 1). A participant may have more than 1 PHC visit during the follow-up period; therefore, a participant can be in more than 1 category or >1 time in the same category which provided 11 613 observations of 6135 unique cases. Home visits were a common visit type (N = 4441) followed by clinic visits (N = 3590). There were 2497 team visits, and 394 visits were of administrative character. There were 691 participants who had no PHC visit.

### PHC Visits Post-Discharge and Hospital Readmission

There was no statistically significant association between number of PHC visits and risk of readmission (Model 1: HR 1.00; 95% CI 1.00-1.01; Table 2). Similarly, there were no statistically significant associations between types of PHC visits and readmission (Model 2: administrative vs no visit HR 0.33, 95%CI 0.08-1.33; clinic visits vs no visit HR 0.93, 95%CI 0.71-1.21; home visits vs no visit HR 1.03, 95%CI 0.84-1.27; team visits vs no visit HR 0.76, 95%CI 0.54-1.07; Table 2).

**Table 2.** Models for Cox Regression With Time Varying Covariate for Association Between Primary Health Care Visits Post-Discharge and Risk of Readmission.

	Readmission in 30 days			
	Unadjusted model		Adjusted* model	
	HR (95% CI)	*P*-value	HR (95% CI)	*P*-value
*Model 1*				
Number of primary health care visits	1.01 (1.00-1.01)	.051	1.00 (1.00-1.01)	.324
*Model 2*				
Type of primary health care visits				
No primary health care visit	Ref.		Ref.	
Administrative work related to care	0.54 (0.20-1.47)	.229	0.33 (0.08-1.33)	.118
Clinic visits	0.88 (0.70-1.11)	.273	0.93 (0.71-1.21)	.588
Home visits	0.99 (0.82-1.19)	.888	1.03 (0.84-1.27)	.771
Team visits	0.76 (0.56-1.02)	.072	0.76 (0.54-1.07)	.115

Abbreviations: HR, hazard ratio; CI, confidence interval.

* Adjusted for all control variables (age, sex, education, living arrangements, number of diagnoses, polypharmacy (≥5 drugs), activity of daily living, risk of malnutrition, risk of fall, risk of pressure ulcer, length of stay of index admission, number of specialist care visits, and home help receipt.

## Discussion

This study explored the associations between PHC visits post-discharge and hospital readmission in 30 days after geriatric inpatient care. The analytic strategy accounted for biases related to exposure time and took into account change in type of visit during the follow-up by modeling PHC visits as time-varying covariates. There were no statistically significant associations between PHC visits post-discharge and the risk of subsequent hospital readmission.

The lack of association between higher utilization of PHC and readmission contradicts a study on heart failure patients perhaps due to the patient characteristics and the measurement of intensity of visits in terms of cost rather than number of visits.^1^ Previous research on emergency care use has shown that frequent users of PHC are also frequent users of emergency care and commonly report unmet needs of PHC.^26^ Early discharge and the complexity of medical conditions that are difficult to manage by the PHC contribute to the risk of readmission.^27^ In our study, analyses were adjusted for potential health status variables; however, register data is too limited to provide information on the clinical complexity of a disease. Moreover, the measurement “number of visits” do not provide details on the components of care received and whether these components were tailored to the needs of patients which can potentially reduce the risk of readmission. Family involvement in the discharge process or family presence in the PHC visits play an important role in facilitating older patients’ engagement in the care process and in the reduction of readmission.^28-30^ However, the impact of family involvement on the association between PHC visits post-discharge and readmission was not explored due to the unavailability of such data. This suggests future studies including factors that are not available in register data such as clinical complexity of a disease, characteristics of treatment received, and role of family.

Research has mainly focused on a single type of PHC visit post-discharge which limits comparisons with previous studies. A specific type of PHC visit has its own potential benefits, for example, home visits post-discharge can assist patients in transitioning from hospital to home by providing care to the patient in their familiar environment and identifying potential barriers to the compliance of medication.^11,15^ The lack of association between types of PHC visits and readmission in our study echoes the findings from studies on home visit^11^ and team visits post-discharge and readmission among individuals discharged home.^13^ Previous research has also shown lack of associations between team-based PHC visits and hospitalization and associated costs among older or chronically ill patients.^31^ Lack of studies including all types of PHC visits limit discussion on whether a particular type of visit or combination of types benefit various group of patients. Moreover, there is no consensus regarding the time frame for measuring care utilization post-discharge visits and readmission.^16^ Survey and qualitative studies could provide insight into the analytical time frame and factors that are relevant in exploring the associations between PHC visits post-discharge and readmission among patients discharged home.

### Strengths and Limitations

A strength of this study was that it was based on register data. Registers provide high quality information on all the PHC visits, hence minimal risk of selection bias- a problem common to surveys. This study accounted for all PHC visits irrespective of care providers’ profession or type of visit. PHC visits were modeled as a time varying covariate, a method that accounts for changes in the covariate status during the follow-up period and explore association between the current value of the covariate prior to the event and the actual event.^32^

This study has several limitations, including that the study sample represents only 3 inpatient geriatric departments in Region Stockholm that may influence the generalizability of findings. The study cohort shows statistically significant differences from the regional cohort in administrative work related to care and number of visits to the speech therapist.^14^ This problem is minor, though, the proportional differences were very small. Although sample size is large, the hospital readmission rate within 30 days might have influenced statistical power in the analysis of PHC visit types. Analyses were adjusted for specialist care visits and home help services for older adults, but unavailability of data limited us from accounting for informal care factors that are relevant to cover full scope of post-discharge care. Complexity of health condition and health care utilization prior to the index admission were not measured due to the unavailability of data which might have biased our results in any direction.

## Conclusions

We found that there were no statistically significant associations between PHC visits post-discharge and readmission. Exposure time, disease complexity, and analytical time frame of study are several challenges in observational studies on care post-discharge and outcomes that should be considered in the interpretation of findings. The political agenda with deliberate reductions in hospital care and reinforcement of PHC implies the need for future studies exploring unmet needs of health care and risk of readmission after geriatric care.
